# ARNTL2 promotes pancreatic ductal adenocarcinoma progression through TGF/BETA pathway and is regulated by miR-26a-5p

**DOI:** 10.1038/s41419-020-02839-6

**Published:** 2020-08-10

**Authors:** Zhifang Wang, Tingting Liu, Wenhua Xue, Yuanyuan Fang, Xiaolong Chen, Lijun Xu, Lixia Zhang, Kelei Guan, Juntao Pan, Lili Zheng, Guijun Qin, Tingting Wang

**Affiliations:** 1 Endocrinology Department, The First Affiliated Hospital of Zhengzhou University, Zhengzhou, 450052 China; 2 Key Laboratory of Clinical Medicine, The First Affiliated Hospital of Zhengzhou University, Zhengzhou, 450052 China; 3 Endocrinology Department, Henan Provincial People’s Hospital, People’s Hospital of Zhengzhou University, Zhengzhou, 450003 China; 4 General Geriatrics, The First Affiliated Hospital of Zhengzhou University, Zhengzhou, 450052 China

**Keywords:** Cell growth, Cancer genetics

## Abstract

Pancreatic ductal adenocarcinoma (PDAC) is one of the most aggressive malignancies and the therapeutic outcomes remain undesirable. Increasing evidence shows that aryl hydrocarbon receptor nuclear translocator like 2 (ARNTL2) plays crucial roles in tumorigenesis of multiple tumors. However, the expression status and functions of ARNTL2 in PDAC remain elusive. Here we showed that ARNTL2 expression was markedly upregulated in PDAC tissues and cell lines. elevated expression of ARNTL2 was positively related to unfavorable prognosis. Knockdown of ARNTL2 could suppress motility and invasive ability of PDAC cells in vitro, as well as tumor development in vivo. In addition, microRNA-26a-5p (miR-26a-5p) was identified as the crucial specific arbitrator for ARNTL2 expression and the expression of miR-26a-5p was inversely correlated with ARNTL2 expression in PDAC tissues. Functionally, elevated expression of miR-26a-5p was found to inhibit the proliferation, migration, and invasion of PDAC cells in vitro, while ARNTL2 increased expression could partially abolish the suppressive effect of miR-26a-5p. Mechanism study indicated that elevated expression of miR-26a-5p suppressed TGF/BETA signaling pathway by targeting ARNTL2 in PDAC cells. In conclusion, our data suggested that ARNTL2 acted as an oncogene to regulate PDAC growth. MiR-26a-5p/ARNTL2 axis may be a novel therapeutic candidate target in PDAC treatment.

## Introduction

Pancreatic ductal adenocarcinoma (PDAC) is one of the most aggressive digestive malignancies in human, which accounts for 95% of all pancreatic cancer^1^. Though many advanced therapeutic strategies were applied, the prognosis of PDAC was far from satisfactory due to its early metastases and recurrences, with only an 8% 5-year overall survival rate (OS)^2,3^. Thus, identification of a novel biomarker with early diagnosis and prognostic significance in PDAC is urgent.

ARNTL2 belongs to the PAS (PER, ARNT, SIM) superfamily encoding a basic helix–loop–helix transcription factor. This protein plays important roles as a biologically relevant partner of circadian and hypoxia factors^4^. An increasing number of evidences have validated the correlation between ARNTL2 and human cancers including lung adenocarcinoma^5^, colorectal cancer^6^, and breast cancer^7^. However, the clinical values and biological roles of ARNTL2 in PDAC have not been reported.

MiRNAs are short, non-coding RNAs which play crucial roles in regulating gene expression mainly through binding to the 3′ untranslated region (3′UTR) of target mRNAs^8,9^. A growing body of evidence suggests that miRNAs are important tumor regulators and play a critical role in the tumorigenesis^10^. They have been emerged as novel therapeutic targets to control the initiation and progress of cancers^11,12^. MiR-26a-5p was reported to exhibit tumor-suppressive properties in multiple cancers^13–15^. However, the expression profiling and function role of miR-26a-5p in PDAC has not been fully documented.

In the present study, ARNTL2 was highly expressed in PDAC tissues and cell lines and high level of ARNTL2 was associated with poor prognosis of PDAC patients. Functional experiments elucidated that ARNTL2 acted as an oncogene in PDAC. Furthermore, we identified the ARNTL2 as a novel target of miR-26a-5p. In addition, the ectopic expression of ARNTL2 could partially reversed the inhibitory effects on cell malignant phenotypes caused by miR-26-5p elevated expression. Mechanistically, we indicated that miR-26a-5p/ARNTL2 axis was critical for initiation and progression of PDAC by regulating TGF/BETA pathway. Together, our findings provided innovative insights for the mechanism research and treatment progress for PDAC.

## Results

### ARNTL2 is highly expressed in PDAC tissues and closely correlated with unfavorable prognosis in ZZU cohort

**Fig. 1:**
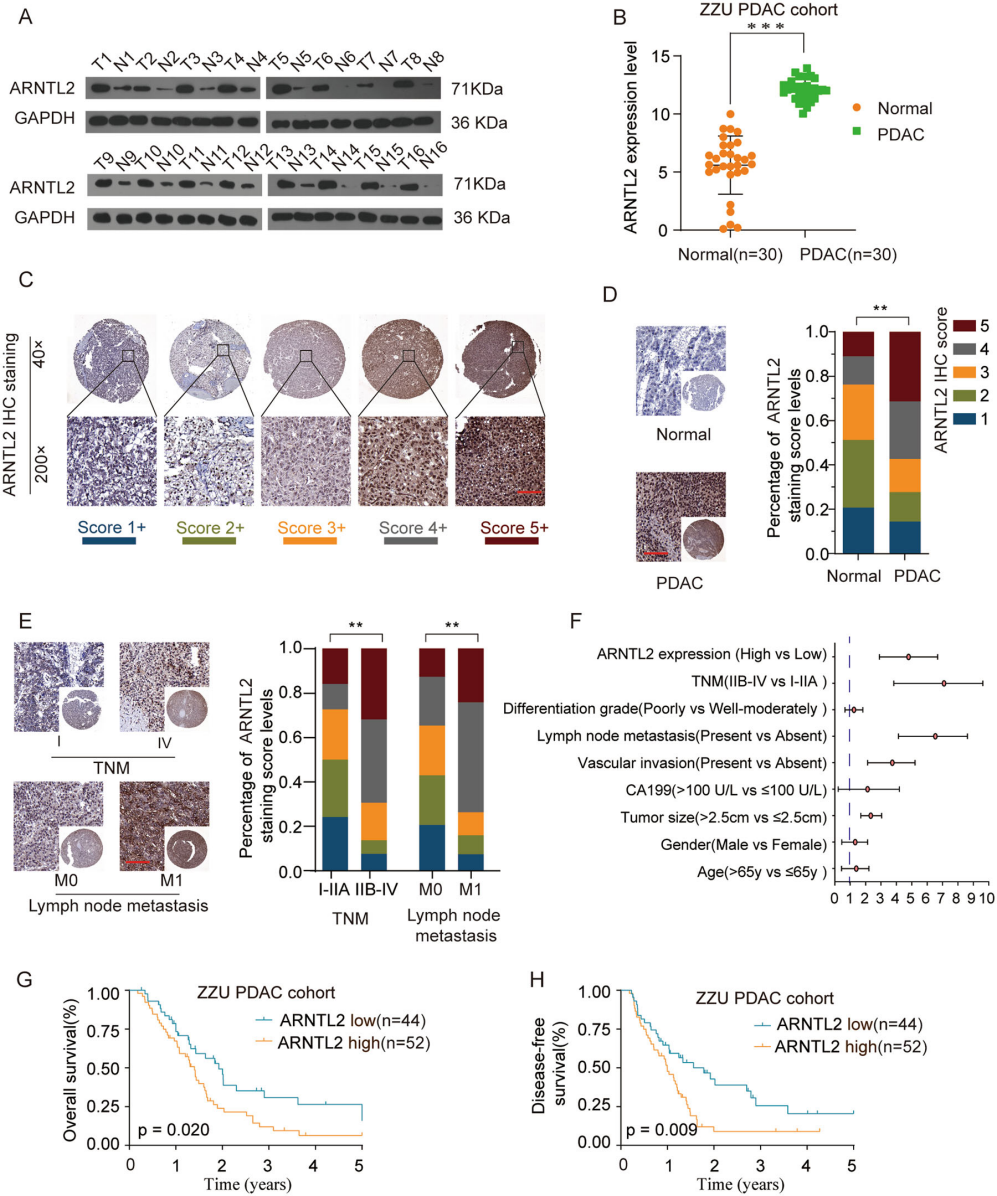
Elevated expression of ARNTL2 correlates with clinicopathological features and unfavorable prognosis in PDAC patients. **a** Western blot analysis was performed for determining expression levels of ARNTL2 in 16 paired PDAC tissues. **b** Paired T test was carried out for ARNTL2 expression in PDAC and paired adjacent normal tissues in ZZU cohort. **c** Representative IHC staining and scoring of ARNTL2 in PDAC TMA cohort. Scale bars, 200 μm. **d** Representative ARNTL2 IHC staining results and the percentage of each ARNTL2 IHC score in PDAC tumor tissues and paired adjacent normal tissues. **e** Representative ARNTL2 IHC staining and percentage of each ARNTL2 IHC score in PDAC with different TNM stages, with or without lymph node metastasis. **f** The multivariate Cox regression analysis were performed to depict the correlations between the clinicopathological features and OS. **g**, **h** Kaplan–Meier survival analysis of the correlation between ARNTL2 expression and OS (**g**), DFS (**h**) of PDAC patient in ZZU cohort. **P < 0.01, ***P < 0.001.

The relative expression level of ARNTL2 was measured in 16 paired PDAC patients’ tissues by western blot. The results suggested that ARNTL2 was highly expressed in PDAC tissues in comparison with surrounding non-tumor tissues (Fig. 1a). To elucidate the expression status of ARNTL2 in PDAC, the expression profile of ARNTL2 was examined by qRT-PCR in 30 paired PDAC patients’ tissues. As shown in Fig. 1b, ARNTL2 mRNA was significantly highly expressed in ARNTL2 patients. Simultaneously, IHC was performed on our PDAC tissue microarray (TMA) cohort.

We used a scoring system based on the different percentage of positively stained cells and staining intensity. The expression levels of ARNTL2 were scored between 1+ to 5+ (Fig. 1c). In addition, the IHC staining results confirmed the elevated ARNTL2 expression level in ARNTL2 (Fig. 1d). We then analyzed the relationship between ARNTL2 staining intensive and clinicopathological characteristics. The results showed that ARNTL2 expression levels were closely associated with TNM stage, lymph node metastasis, tumor size, and vascular invasion (Fig. 1e and Table 1). Furthermore, ARNTL2 expression was found to be independent prognostic factors by univariate and multivariate analysis (Fig. 1f and Table 2). Furthermore, univariate and multivariate analyses showed that advanced TNM stages, lymph node metastasis, vascular invasion, and high ARNTL2 expression were proved independent prognostic factors (Fig. 1f and Table 2). Kaplan–Meier analysis also demonstrated that overall survival rate (OS) and disease-free rate (DFS) were markedly reduced in PDAC afflicted patients with higher ARNTL2 expression (Fig. 1g, h). Overall, these data showed that elevated ARNTL2 expression is strongly associated with unfavorable prognosis in PDAC patients.

**Table 1:** Correlation of clinic-pathological features with ARNTL2 expression in PDAC TMA corhort.

Clinicopathological features variables		ARNTL2 expression		*P* value
		Low expression (*n* = 44) High expression (*n* = 52)		
Age (years)	≤65	16 (36.4%)	27 (51.9%)	0.186
	>65	28 (63.6%)	25 (48.1%)	
Gender	Female	14 (31.8%)	22 (42.3%)	0.397
	Male	30 (68.2%)	30 (57.7%)	
CA199	≤100 U/L	30 (68.2%)	25 (48.1%)	0.076
	>100 U/L	14 (31.8%)	27 (51.9%)	
Lymph node metastasis	Absent	27 (61.4%)	14 (26.9%)	**0.001**
	Present	17 (38.6%)	38 (73.1%)	
Vascular invasion	Absent	26 (59.1%)	19 (36.5%)	**0.045**
	Present	18 (40.9%)	33 (63.5%)	
Tumor size	≤2.5 cm	12 (27.3%)	32 (61.5%)	**0.002**
	>2.5 cm	32 (72.7%)	20 (38.5%)	
TNM stage	I–IIA	27 (61.4%)	16 (30.8%)	**0.005**
	IIB–IV	17 (38.6%)	36 (69.2%)	
Differentiation grade	Well-moderately	30 (68.2%)	28 (53.8%)	0.222
	Poorly	14 (31.8%)	24 (46.2%)	

Bold values indicate statistical significance, *P* < 0.05.

**Table 2:** Correlation of clinic-pathological features with ARNTL2 expression in PDAC cohort.

	Univariate analysis			Multivariate analysis		
	HR	95% CI	*P* value	HR	95% CI	*P* value
*Univariate and multivariate analysis of overall survival in PDAC patients* (*n* = *96)*						
Age (>65 vs ≤65)	1.527	0.436–2.220	0.184			
Gender (male vs female)	1.382	0.456–2.130	0.212			
CA199 (>100 U/L vs ≤100 U/L)	1.972	0.225–4.192	0.165			
Lymph node metastasis (present vs absent)	6.841	4.133–8.623	**0.002**	6.352	3.732–9.209	**0.004**
Vascular invasion (present vs absent)	3.863	2.136–5.217	**0.015**	3.785	2.056–5.391	**0.020**
Tumor size (>2.5 cm vs ≤ 2.5 cm)	2.274	1.693–3.044	**0.021**	1.661	0.441–2.880	0.168
TNM stage (IIB–IV vs I–IIA)	7.841	3.833–9.623	**0.001**	6.968	3.501–9.326	**0.002**
Differentiation grade (poor vs well)	1.143	0.296–2.292	0.202			
ARNTL2 expression (high vs low)	5.565	1.197–7.611	**0.005**	5.516	1.532–7.411	**0.011**
*Univariate and multivariate analysis of disease-free survival in PDAC patients (n* = *96)*						
Age (>65 vs ≤65)	1.052	0.823–1.232	0.302			
Gender (male vs female)	0.871	0.672–1.035	0.415			
CA199 (>100 U/L vs ≤100 U/L)	0.925	0.76–1.103	0.381			
Lymph node metastasis (present vs absent)	5.235	2.029–7.321	**0.004**	5.950	2.208–7.733	**0.002**
Vascular invasion (present vs absent)	4.022	1.628–6.256	**0.012**	3.627	1.756–5.862	**0.017**
Tumor size (>2.5 cm vs ≤ 2.5 cm)	2.329	1.558–3.717	**0.025**	2.361	0.372–4.013	0.156
TNM stage (IIB–IV vs I–IIA)	6.182	3.486–8.962	**0.002**	7.675	3.653–9.554	**0.000**
Differentiation grade (poor vs well)	2.021	0.512–4.205	0.140			
ARNTL2 expression (high vs low)	4.625	2.031–6.025	**0.008**	4.432	2.017–5.981	**0.010**

Bold values indicate statistical significance, *P* < 0.05.

### Elevated ARNTL2 in public databases and correlated with unfavorable prognosis in PDAC patients

**Fig. 2:**
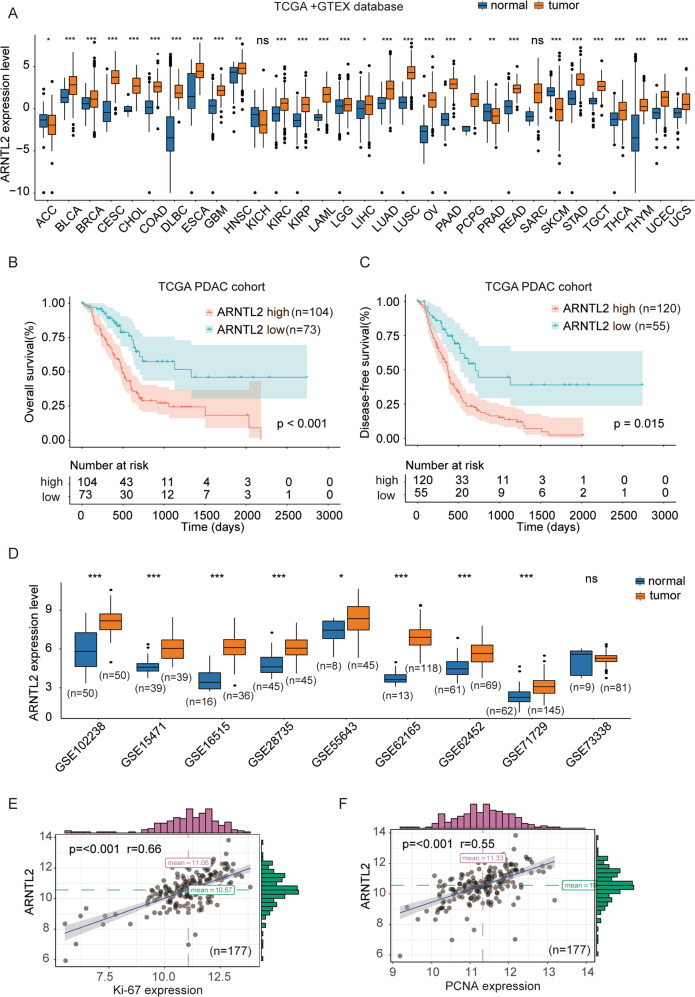
High expression of ARNTL2 indicates poor prognosis of patients with PDAC. **a** ARNTL2 was frequently highly expressed in most cancers in TCGA and GTEX database by bioinformatics analysis. **b**, **c** Kaplan–Meier survival analysis of the positive correlation between ARNTL2 expression and OS (**b**) or DFS (**c**) in TCGA PDAC cohort. **d** The elevated expression levels of ARNTL2 in PDAC compared with normal tissues were analyzed in nine independent GEO cohorts. **e**, **f** The positive correlation of ARNTL2 expression with Ki67 and PCNA expression in TCGA PDAC cohort. *P < 0.05, **P < 0.01, ***P < 0.001.

To explore ARNTL2 expression pattern in common cancers, expression levels of ARNTL2 in various types of cancers were analyzed using The Cancer Genome Atlas (TCGA) and Genotype-Tissue Expression (GTEX) database. The results indicated that ARNTL2 was frequently highly expressed in most cancers (Fig. 2a). Similar with the results obtained based on TMA analysis. Kaplan–Meier analysis indicated that patients in the ARNTL2-high group had a shorter OS rate (Fig. 2b; *P* < 0.001) and DFS rate (Fig. 2c; *P* = 0.015) than that of the ARNTL2-low group in the TCGA PDAC cohort. In addition, we confirmed the elevated expression of ARNTL2 in PDAC tissues compared with normal tissues in 9 independent GEO datasets (Fig. 2d). Meanwhile, ARNTL2 expression was positively associated with the expression of proliferation marker Ki67 and PCNA (Fig. 2e, f). Taken together, above findings provided evidences indicating a closely correlation between ARNTL2 expression and PDAC progression.

### Knockdown of ARNTL2 inhibits proliferation, migration, and invasion of PDAC cells in vitro

**Fig. 3:**
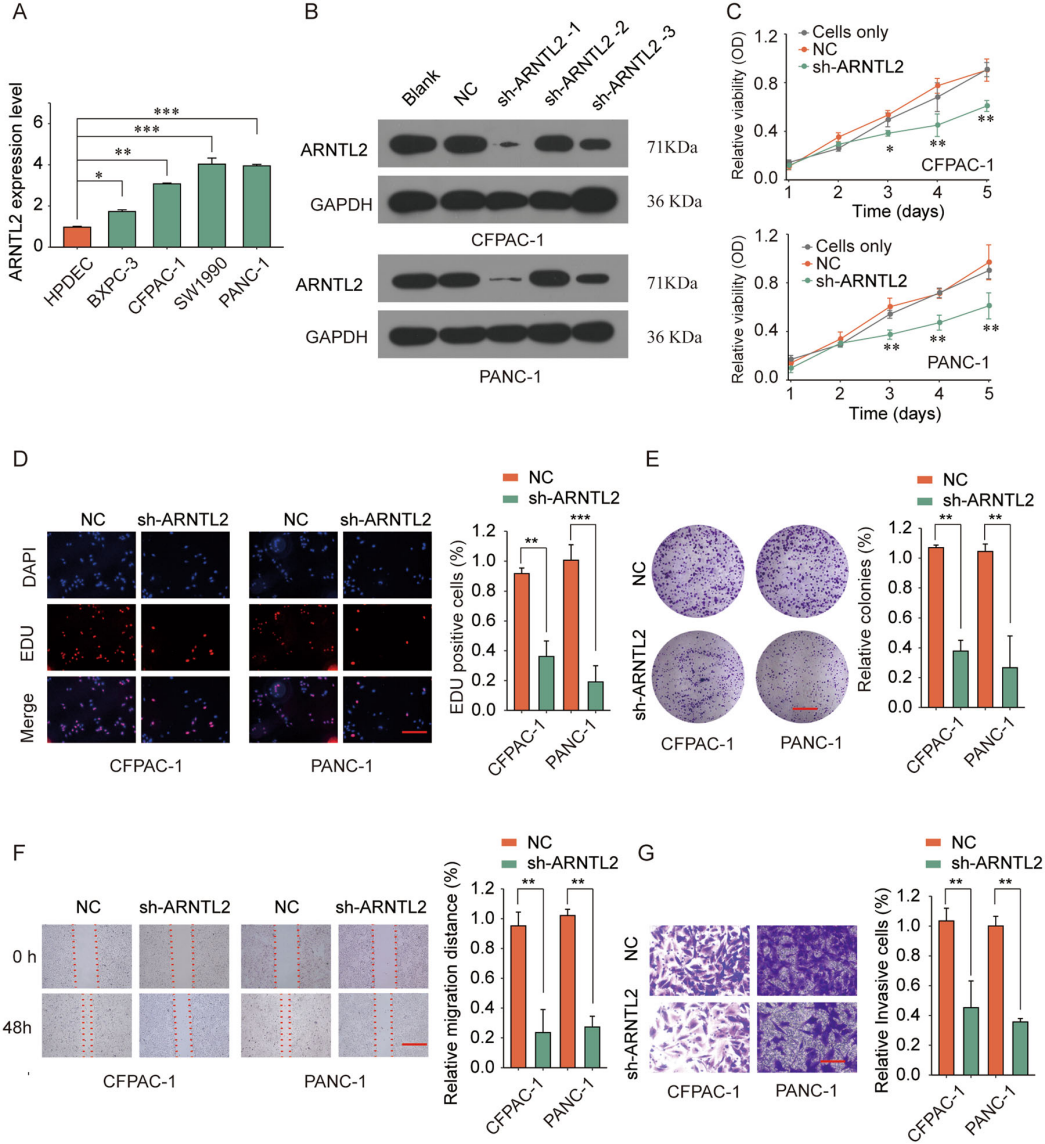
Downregulation of ARNTL2 inhibits PDAC cell growth, migration, and invasion in vitro. **a** The elevated mRNA expression levels of ARNTL2 in PDAC cell lines (BXPC-3, CFPAC-1, SW1990, and PANC-1) and normal pancreas cells (HPDEC) were determined by qRT-PCR analysis. **b** The knockdown efficiency was evaluated by western blot analysis. **c**–**e** Cell proliferation capacity was analyzed by CCK-8 assay (**c**), EDU staining assay, scale bars, 50 μm (**d**), and colony formation assay, scale bars, 8 mm (**e**), which revealed that knockdown of ARNTL2 dramatically inhibited PDAC cells proliferation, DNA synthesis and colony formation. **f** Wound-healing assay determined the effect of ARNTL2 on cell migration capability. Scale bars, 500 μm. **g** The invasion capability of PDAC cells transfected with NC or sh-ARNTL2 was analyzed by transwell assay. Scale bars, 50 μm. *P < 0.05, **P < 0.01, ***P < 0.001. The above ex*p*eriments proved that silencing ARNTL2 could also inhibit the migration and invasion capacity of PDAC cells.

To investigate the biological function of ARNTL2 in PDAC cells, we determined the ARNTL2 expression in normal pancreas cells (HPDEC) and PDAC cell lines (BXPC-3, CFPAC-1, SW1990, and PANC-1). ARNTL2 expression was elevated expressed in PDAC cells compared with normal pancreas cells (Fig. 3a). The results of western blot assay were almost consistent (Supplementary Fig. S1A). Next, we used lentivirus to knock down ARNTL2 expression in CFPAC-1 and PANC-1 cell lines. Western blot was performed to confirm the transfection efficiency (Fig. 3b). Immunofluorescence assay confirmed the significantly decreased expression of ARNTL2 after transfection with ARNTL2 shRNA (Supplementary Fig. S1B). Moreover, CCK-8 assay (Fig. 3c), EDU staining assay (Fig. 3d), and colony formation assay (Fig. 3e) revealed that knockdown of ARNTL2 dramatically inhibited PDAC cells proliferation, DNA synthesis and colony formation, respectively, in comparison with that in PDAC cells transfected with NC control. In addition, silencing ARNTL2 could also inhibit the migration and invasion capacity of PDAC cells (Fig. 3f, g). On the contrary, overexpression of ARNTL2 promotes PDAC cell proliferation and metastasis in vitro (Supplementary Fig. S2). Furthermore, the result of Tunel assay demonstrated that cell apoptosis rate was promoted after ARNTL2 knockdown (Supplementary Fig. S1C), which was further confirmed by western blot assay in apoptosis related proteins (caspase-3, cleaved caspase-3, Bax, Bak, Bcl-2) (Supplementary Fig. S1D). Taken together, these data exhibited that ARNTL2 might be a key promoter of PDAC cell growth and invasion.

### Knockdown of ARNTL2 suppresses tumor growth in vivo

**Fig. 4:**
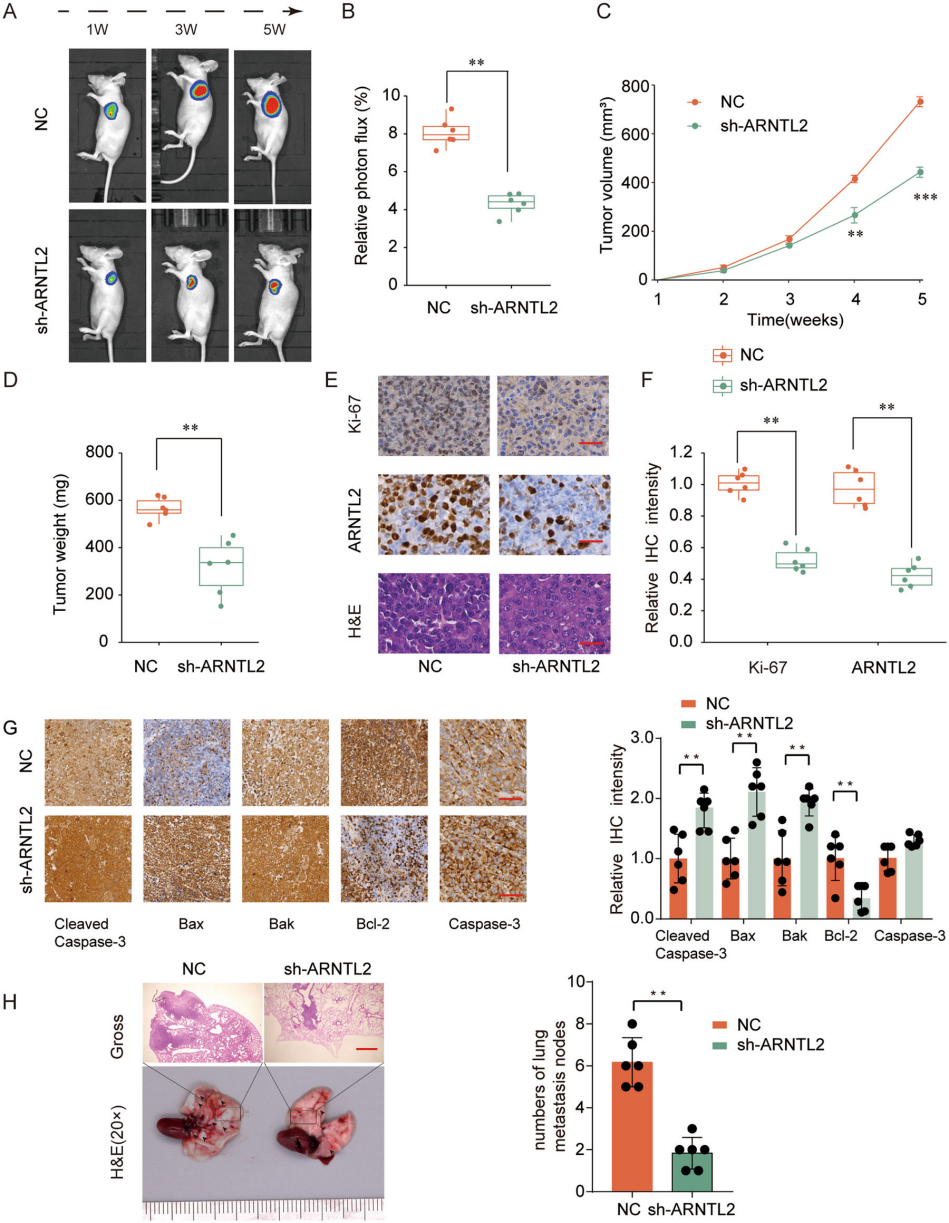
Knockdown of ARNTL2 represses tumor growth and ki-67 expression in PDAC xenografts. **a** Representative images of nude mice. **b** Relative photon flux were quantified using the IVIS imaging system at week 5, which showed that the ARNTL2-depleted group (sh-ARNTL2) mice developed weaker luciferase signal compared with the control group (NC). **c**, **d** Similar results in quantitative analysis of xenografted tumor volume (**c**) and weight (**d**). **e** Representative H&E staining and Ki67, ARNTL2 IHC staining in PDAC xenografts after sh-ARNTL2 treatment. Scale bars, 200 μm. **f** Relative IHC expression levels of ARNTL2 and Ki67 were obviously decreased in sh-ARNTL2 group. **g** IHC analysis of Bcl-2, Bax, Bak, cleaved caspase-3, and caspase-3 proteins in tumors. **h** Representative results of gross and H&E staining of metastatic lung nodules, sh-ARNTL2 treatment decreased lung metastasis occurrence. Scale bars, 200 μm. **P < 0.01, ***P < 0.001.

To verify the effect of ARNTL2 on PDAC tumorigenesis in vivo, we transduced lentivirus to stably knock down ARNTL2 (sh-ARNTL2) or corresponding control (NC) in PANC-1 cells. The ARNTL2-depleted group (sh-ARNTL2) mice developed weaker luciferase signal compared with the control group (NC) (Fig. 4a, b). Consistently, tumor volume and weight were noticeably lower in the ARNTL2 silencing group compared with those in NC group (Fig. 4c, d). In addition, IHC staining intensive of ARNTL2 and the proliferation marker Ki67 were obviously decreased in sh-ARNTL2 group (Fig. 4e, f). Meanwhile, we observed a decrease in the anti-apoptotic Bcl-2, and an increase in the pro-apoptotic Bax and Bak proteins, with an increased level of cleaved caspase-3 in sh-ARNTL2 group (Fig. 4g). As shown in Fig. 4h, sh-ARNTL2 treatment remarkably reduced the metastatic potential of PANC-1 cells, as evidenced by the decreased lung metastasis occurrence. Altogether, these results strongly indicate that ARNTL2 knockdown significantly restrains the tumorigenesis of PDAC.

### ARNTL2 regulates TGF/BETA signaling pathway in human PDAC

**Fig. 5:**
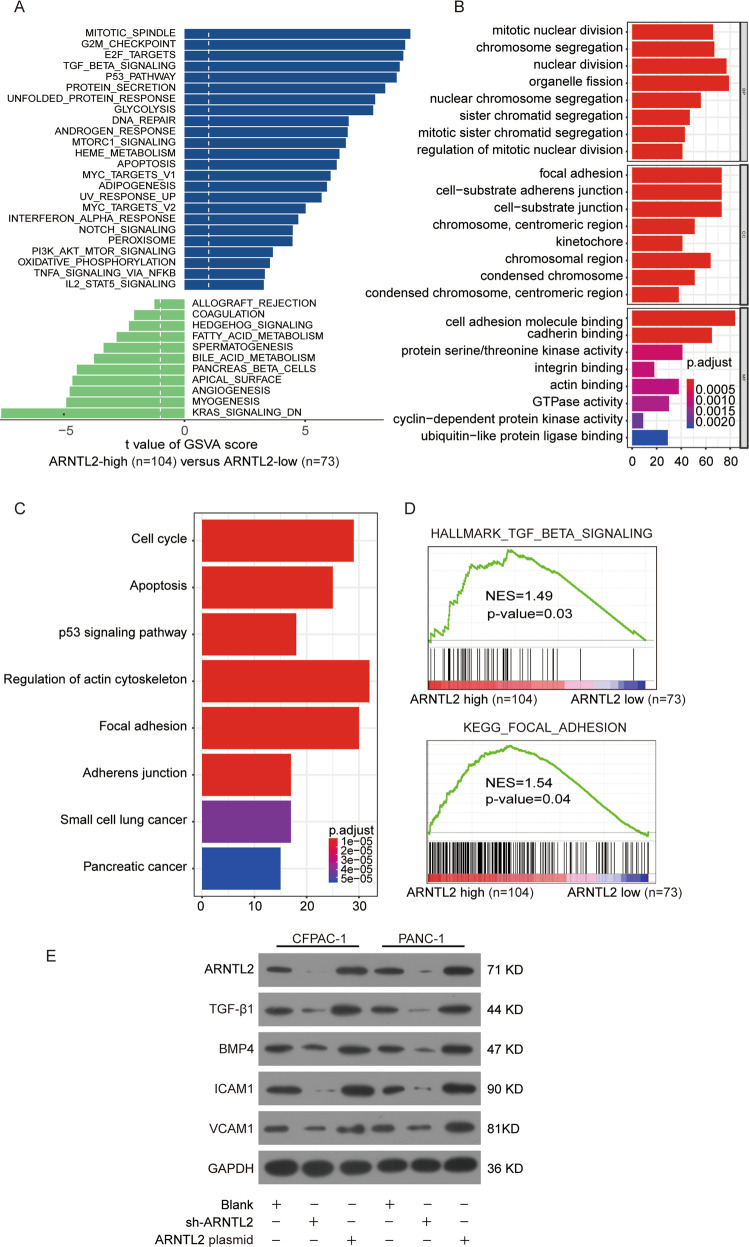
ARNTL2 was positively correlated with TGF/BETA signaling pathway and cell focal adhesion molecules. **a** GSVA analysis showed the activated pathway in tumor compared with adjacent non-tumor in TCGA PDAC cohort. TGF/BETA signaling pathway might be associated with elevated ARNTL2 expression. **b**, **c** KEGG (**b**) and GO (**c**) analysis showed the enriched biological functions of differentially expressed gene profiles, indicated that the focal adhesion served as one of the major enriched signaling with high ARNTL2 expression. **d** GSEA analysis identified the enrichment of TGF/BETA signaling pathway and focal adhesion in TCGA PDAC cohort. **e** Western blot analysis identified that the protein levels of TGF/BETA signaling pathway and cell focal adhesion related molecules in CFPAC-1 or PANC-1 cells were increased in ARNTL2-overexpressed group but were decreased in ARNTL2-silenced group.

To further explore the potential mechanisms that ARNTL2 may regulate in PDAC, we performed bioinformatics analysis based on PDAC TCGA cohort. Gene Set Variation Analysis (GSVA) revealed that the markedly correction between the ARNTL2 expression and the activation of TGF/BETA pathway (Fig. 5a). Kyoto Encyclopedia of Genes and Genomes (KEGG) and Gene Ontology (GO) indicated that focal adhesion was markedly enriched in ARNTL2-high expression samples (Fig. 5b, c), indicating that ARNTL2 might contribute to PDAC development through regulating cell focal adhesion. Consistently, Gene Set Enrichment Analysis (GSEA) plots demonstrated that ARNTL2 expression was closely correlated with TGF/BETA signaling pathway and cell focal adhesion (Fig. 5d). Furthermore, we demonstrated that the expression levels of TGF/BETA and cell focal adhesion pathway associated proteins, such as TGF-β1, BMP4, ICAM1, and VCAM1 were dramatically increased in ARNTL2-overexpressed group but were decreased in ARNTL2-silenced group (Fig. 5e). Taken together, these findings indicated that the potential mechanism of ARNTL2 was associated with focal adhesion, and TGF/BETA pathway was down-stream of ARNTL2.

### MiR-26a-5p directly targets ARNTL2 in PDAC cells

**Fig. 6:**
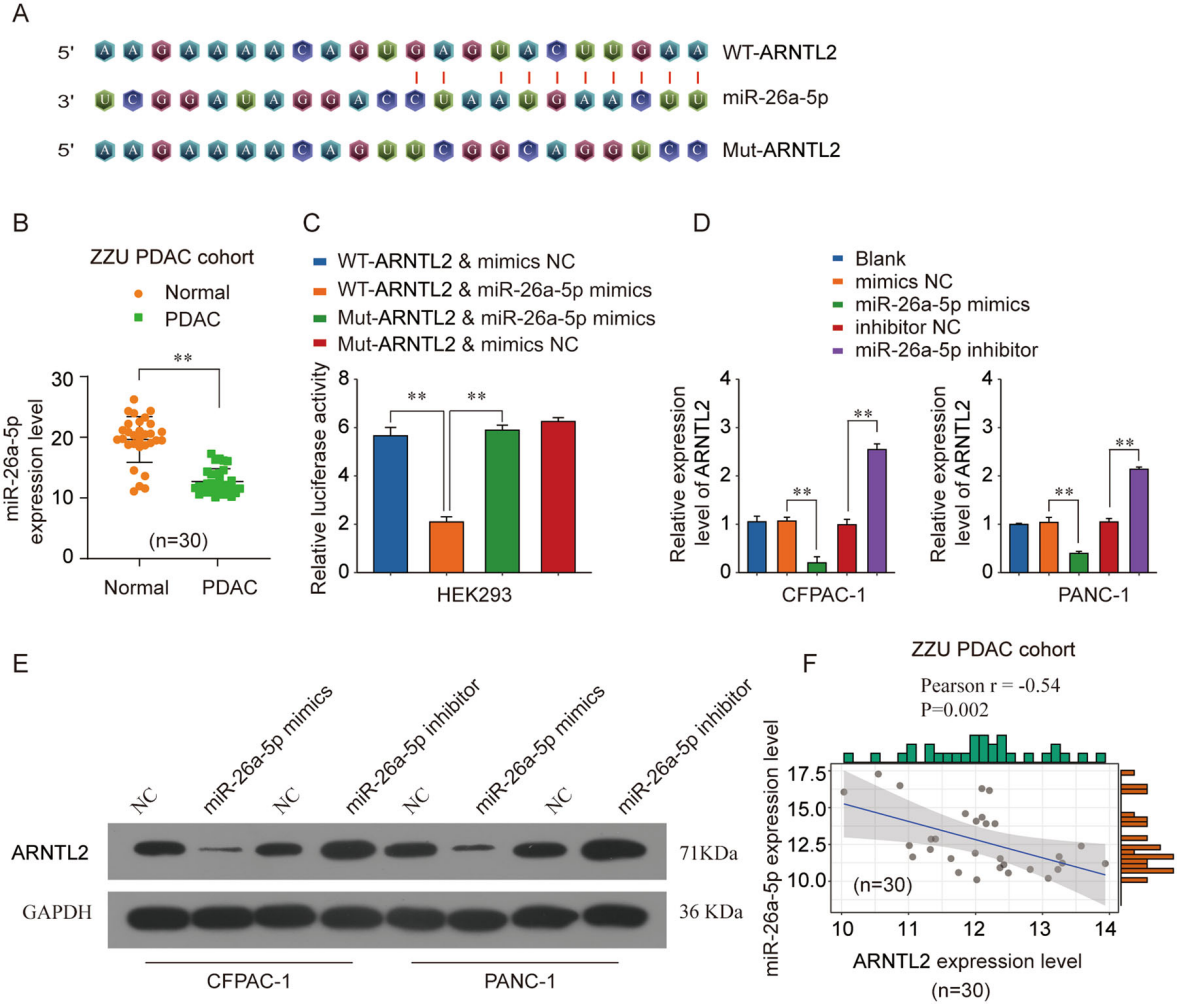
Identification of ARNTL2 as a miR-26a-5p target in PDAC. **a** Predicted binding sites of ARNTL2 WT 3′-UTR or ARNTL2 Mut 3′-UTR to miR-26a-5p. **b** The decreased expression levels of miR-26a-5p in the PDAC samples than that in adjacent non-tumor tissues by qRT-PCR analysis in ZZU cohort. **c** MiR-26a-5p mimics and ARNTL2 3′-UTR reporter wild-type (WT) or mutated (MT) were co-transfected in HEK293 cells. Relative dual-luciferase activity was measured 48 h after transfection by illuminometer. The mRNA expression (**d**) and protein expression levels (**e**) of ARNTL2 in CFPAC-1 or PANC-1 cells were detected by qRT-PCR or western blot assays. **f** The negatively correlation between ARNTL2 expression level and miR-26a-5p expression level from ZZU cohort was analyzed. **P < 0.01.

To investigate the underlying molecular mechanisms of ARNTL2 dysregulation in PDAC progression. We performed bioinformatics analysis to predict the potential miRNAs targeting ARNTL2. MiR-26a-5p was predicted to have putative ARNTL2 binding sites (Fig. 6a). Meanwhile, the decreased expression of miR-26a-5p was found in our own fresh PDAC tissues by qRT-PCR analysis compared with that in normal tissues (Fig. 6b). To test whether ARNTL2 is a direct target of miR-26a-5p, a luciferase activity assay was performed. Overexpression of miR-26a-5p significantly decreased luciferase activity of reporter gene with wild-type ARNTL2–3′UTR compared with negative control (Fig. 6c). Furthermore, qRT-PCR analysis of ARNTL2 mRNA levels was performed in CFPAC-1 and PANC-1 cells. The results showed that ARNTL2 mRNA expression were significantly suppressed after transfected with miR-26a-5p mimics, while miR-26a-5p inhibitor enhanced ARNTL2 expression (Fig. 6d). These changes were also observed at the protein levels, as indicated by western blot analysis (Fig. 6e). Indeed, Pearson correlation analysis revealed that miR-26a-5p was negatively correlated with ARNTL2 expression in ZZU PDAC cohort (Fig. 6f). Altogether, our results demonstrate that miR-26a-5p directly targets ARNTL2 through interaction with its 3′-UTR.

### Elevated expression of miR-26a-5p inhibits PDAC cells proliferation, migration, and invasion by targeting ARNTL2

**Fig. 7:**
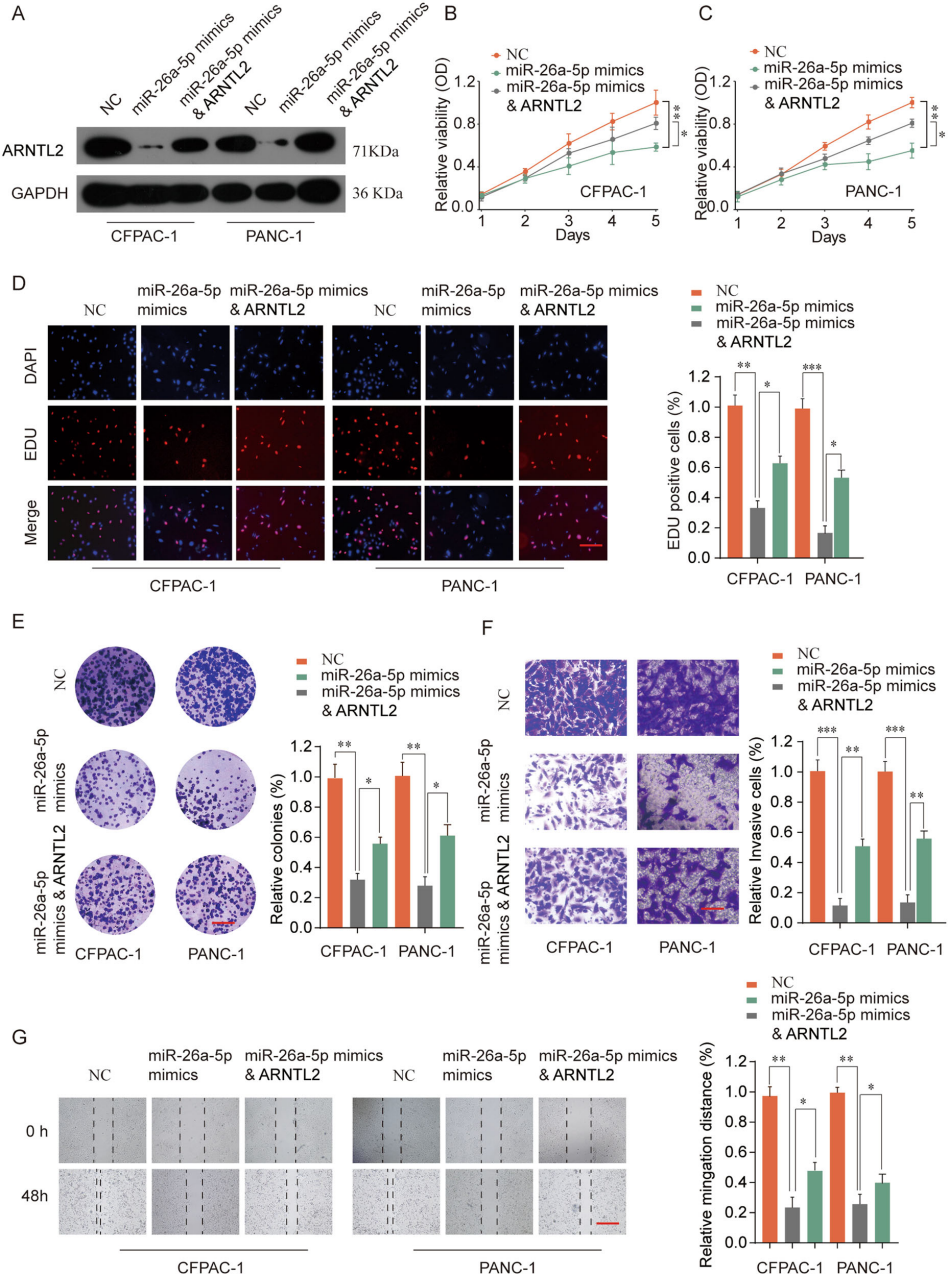
Upregulation of ARNTL2 is crucial for the miR-26a-5p-mediated inhibition of proliferation and migration in PDAC cells. **a** Co-transfection of miR-26a-5p mimics with ARNTL2-overexpression plasmid in CFPAC-1 or PANC-1 cells. The expression levels of ARNTL2 were analyzed by western blot. ARNTL2 plasmid could reverse the inhibition effects of miR-26a-5p on ARNTL2 expression. **b**–**e** CCK-8 assay (**b**, **c**), EDU staining, scale bars, 50 μm (**d**), and colony formation assays, scale bars, 8 mm (**e**) were performed to evaluate cell proliferation of CFPAC-1 or PANC-1 cells. The above functional experiments certified that the inhibitory effect of miR-26a-5p mimics on PDAC cell growth could be partially abolished by ARNTL2 elevated expression. **f** Transwell assay showed that ARNTL2 elevated expression could also partially reverse the inhibitory effect of miR-26a-5p on invasion of PDAC cells. Scale bars, 50 μm. **g** Wound-healing assay determined cell migration capability of CFPAC-1 or PANC-1 cells in different groups. Scale bars, 500 μm. *P < 0.05, **P < 0.01, ***P < 0.001.

To further determine whether miR-26a-5p exerts biological functions by targeting ARNTL2. Initially, miR-26a-5p mimics was transfected into CFPAC-1 and PANC-1 cells co-transfected with ARNTL2-overexpression plasmid or negative control (NC). As shown in Fig. 7a, ARNTL2 protein expression was decreased in miR-26a-5p mimics group, co-transfected with ARNTL2 plasmid and miR-26a-5p mimics could reverse the inhibition effects of miR-26a-5p on ARNTL2 expression. Functional experiments certified that the inhibitory effect of miR-26a-5p mimics on PDAC cell growth could be partially abolished by ARNTL2 elevated expression (Fig. 7b–e). Furthermore, ARNTL2 elevated expression could also partially reverse the inhibitory effect of miR-26a-5p on invasion and migration of PDAC cells (Fig. 7f, g). Thus, these findings reinforced that miR-26a-5p might exert its function via regulating ARNTL2 expression.

### MiR-26a-5p/ARNTL2 axis regulates TGF/BETA signaling in human PDAC

**Fig. 8:**
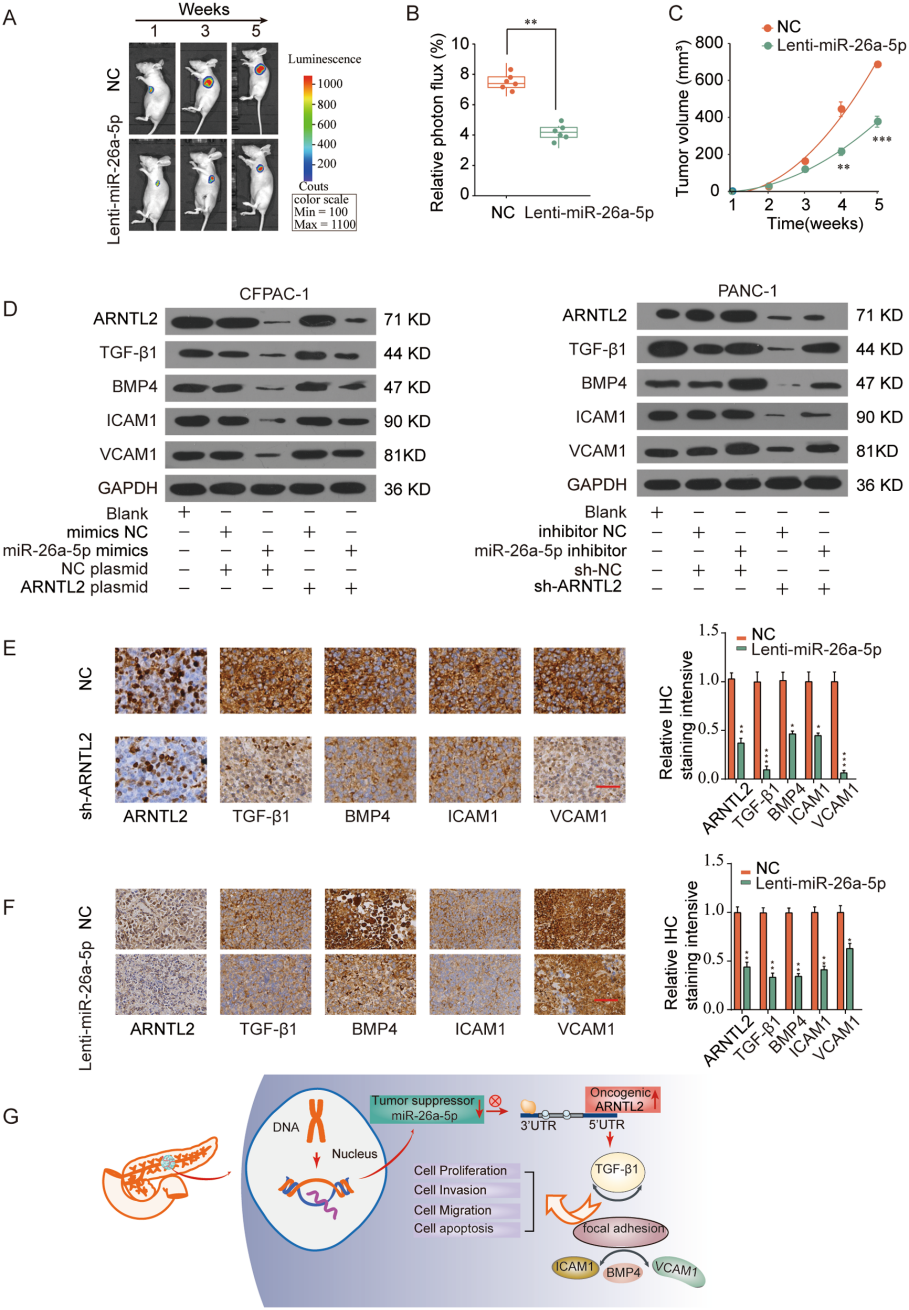
MiR-26a-5p/ARNTL2 axis regulates TGF/BETA signaling pathway and cell focal adhesion in PDAC. **a** Representative images of nude mice. **b** Relative photon flux were quantified using the IVIS imaging system at week 5, which showed that the Lenti-miR-26a-5p group developed weaker luciferase signal compared with the control group (NC). **c** Similar results in quantitative analysis of xenografted tumor volume. **d** Western blot analysis of TGF/BETA signaling pathway and cell focal adhesion related proteins in CFPAC-1 or PANC-1 cells. **e** IHC results revealed that the expression levels of ARNTL2, TGF-β1, BMP4, ICAM1, and VCAM1 in the ARNTL2-knockdown xenograft tumor tissues were decreased. Scale bars, 200 μm. **f** Consistently, the expression levels of ARNTL2, TGF-β1, BMP4, ICAM1, and VCAM1 in the miR-26a-5p overexpression xenograft tumor tissues were analyzed by IHC. Scale bars, 200 μm. **g** Schematic representation depicting the mechanisms that miR-26a-5p/ARNTL2 axis affects the progression of PDAC.

We further evaluated the effects of miR-26a-5p/ARNTL2 axis on TGF/BETA signaling pathway. PANC-1 cells transfected with corresponding control (NC) or miR-26a-5p mimics (Lenti-miR-26a-5p) were subcutaneously implanted into nude mice. Compared with the tumors from the NC group, the Lenti-miR-26a-5p group showed significantly suppressed tumor growth (Fig. 8a–c). Consistently, tumor weight was noticeably lower in Lenti-miR-26a-5p group compared with those in NC group (Supplementary Fig. S3A). In addition, IHC staining intensive of ARNTL2 and the proliferation marker Ki67 were obviously decreased in Lenti-miR-26a-5p group (Supplementary Fig. S3B, C). Western blot assay suggested that the expression levels of TGF/BETA signaling pathway and cell focal adhesion associated proteins, such as TGF-β1, BMP4, ICAM1, and VCAM1 were dramatically decreased in PDAC cells transfected with miR-26a-5p mimics or sh-ARNTL2. Meanwhile, ARNTL2 elevated expression could partially reverse the miR-26a-5p induced inhibitory effect (Fig. 8d). In addition, IHC staining revealed that TGF-β1, BMP4, ICAM1, and VCAM1 expression was decreased in the ARNTL2-knockdown xenograft tumor tissues (Fig. 8e). Consistent results were observed in the miR-26a-5p elevated expression xenograft tumor tissues (Fig. 8f). Subsequently, we performed functional rescued experiments as following: knock down TGF-β1 in ARNTL2-overexpression experiments, while induce overexpression of TGF-β1 in ARNTL2-knockdown experiments. The results indicated ARNTL2 overexpression significantly promoted cell proliferation and invasion of CFPAC-1 and PANC-1 cells, while TGF-β1 silencing antagonized the promoting effects of ARNTL2 plasmid. Furthermore, co-transfection of TGF-β1 overexpression plasmid reverted the suppressive effects of ARNTL2 knockdown on the proliferation and invasion of PDAC cells (Supplementary Fig. S4). Overall, our results suggest that ARNTL2 might exert its function via regulating TGF/BETA pathway. For mechanism exploration, we identified that miR-26a-5p/ARNTL2 axis contributed to the progression of PDAC via activating TGF/BETA signaling pathway and cell focal adhesion (Fig. 8g).

## Discussion

Although substantial progress in the recognizing and therapeutic strategies of PDAC, early metastasis contributes to very few opportunities for radical excision and associates with an extremely poor prognosis in PDAC patients^3,16^. Therefore, further understanding of the genetic changes associated with PDAC progression is essential for improving the treatment and prognosis.

ARNTL2 is widely considered as an important regulator, the oncogenic effects have been reported in many human cancers. Recent study demonstrated that ARNTL2 was a metastasis susceptibility gene for estrogen receptor-negative breast cancer^7^. In addition, Brady et al. reported that ARNTL2 could drive metastatic self-sufficiency and high expression of ARNTL2 predicted poor lung adenocarcinoma patient outcome^5^. Moreover, ARNTL2 was accepted as a potential biomarker for tumor aggressiveness in colorectal cancer^6^. For the first time, we reported the function and potential mechanism of ARNTL2 in PDAC. In the current study, we also revealed that ARNTL2 expression was significantly upregulated in PDAC tissues than the normal tissues, and high level of ARNTL2 was closely associated with aggressive malignant phenotypes and poor survival of PDAC patients. In addition, functional experiments demonstrated that high expression of ARNTL2 facilitated cell proliferative, migration and invasion (Supplementary Fig. S1). Moreover, functional experiments verified that ARNTL2 knockdown impeded PDAC cells’ proliferation and invasion in vitro (Fig. 3), while dampened tumor growth in vivo (Fig. 4). Taken together, above data elucidated that ARNTL2 played oncogenic role in PDAC progression.

A growing body of evidence demonstrated a crucial role for miRNAs in triggering cancer progression and metastasis depend on the targeted mRNAs, including PDAC^17,18^. In our study, we verified that ARNTL2 was a directly functional target of miR-26a-5p. MiR-26a-5p, a classical molecule of miRNA family, has been identified as a tumor suppressor and plays an important role in anti-proliferation and anti-metastasis properties in certain malignancies. In HCC, downregulation of miR-26a-5p promoted tumor metastasis and correlated with poor prognosis^19^. MiR-26a-5p was significantly decreased and promoted proliferation and invasion in prostate cancer by directly targeting SERBP1^20^. In addition, miR-26a-5p was reported to be able to inhibit proliferation and metastasis in papillary thyroid carcinoma through repressing the expression of Wnt5a^21^. Nevertheless, the expression status of miR-26a-5p and its underlying molecular mechanisms in PDAC remained unclear. In this study, we for the first time uncovered that miR-26a-5p was significantly downregulated in PDAC tissues compared with adjacent normal tissues (Fig. 6). Further gain-function experiments confirmed that forced expression of miR-26a-5p inhibited the proliferation, migration and invasion of PDAC cells in vitro, while elevated expression of ARNTL2 could partly reverse the inhibitory effects of miR-26a-5p (Fig. 7). Collectively, these results indicated that miR-26a-5p functioned as tumor suppressor in PDAC via targeting ARNTL2.

Our bioinformatics analysis demonstrated that ARNTL2 elevated expression was positively correlated with the TGF/BETA signaling pathway activation and cell focal adhesion (Fig. 5a, d). Furthermore, western blot assay indicated that the expression levels of TGF/BETA signaling pathway and cell focal adhesion associated proteins, such as TGF-β1, BMP4, ICAM1, and VCAM1 were dramatically increased in ARNTL2-overexpressed group but were decreased in ARNTL2-silenced group (Fig. 5e). Recent literature has documented that TGF/BETA signaling pathway participate in the progression of malignancies^22^. In PDAC, the TGF/BETA signaling pathway has been verified as a promoter of tumor progression^17^. In response to elevated TGF/BETA levels, the bladder cancer cells become more invasive and migratory^23^. Harjunpää et al. have described how malignant cells can utilize cell adhesion molecules to promote tumor growth and metastases^24^. ICAM1 and VCAM1 are two important adhesion molecules, which are considered to be important in the tumorigenesis and progression of malignant tumor^25–28^. In present study, the protein levels of TGF/BETA signaling pathway and cell focal adhesion associated molecules in PDAC cells were significantly decreased after miR-26a-5p elevated expression but were highly expressed when transfected with the ARNTL2 plasmid. Moreover, in the group co-transfected with miR-26a-5p mimics and ARNTL2 plasmid, the inhibitory effects of miR-26a-5p were reversed (Fig. 8a). Taken together, our results suggested that miR-26a-5p/ARNTL2 axis was involved in PDAC progression through controlling TGF/BETA signaling pathway and cell focal adhesion (Fig. 8c).

In summary, we found that ARNTL2 is a novel target of miR-26a-5p. As a tumor promoter in PDAC, knockdown of ARNTL2 arrests tumorigenesis and metastasis via TGF/BETA signaling pathway. These data uncover that miR-26a-5p/ARNTL2 axis may serve as novel biomarkers and therapeutic targets for PDAC.

## Materials and methods

### Human specimens

Totally 96 PDAC tissue specimens and 96 surrounding non-tumorous tissues were collected from the First Affiliated Hospital of Zhengzhou University (Zhengzhou, China). The clinical characteristics of the PDAC patients were listed in Table 1. This study was approved and supervised by the Ethics Committee of the First Affiliated Hospital of Zhengzhou University,

### Dataset acquisition and process

Expression data for ARNTL2 in pan-cancers were explored from TCGA (The Cancer Genome Atlas) and GTEX (Genotype-Tissue Expression) RNAseq database. Nine publicly available independent PDAC microarray datasets (GSE102238, GSE15471, GSE16515, GSE28735, GSE55643, GSE62165, GSE62452, GSE71729, and GSE73338) were extracted from the GEO (Gene Expression Omnibus) database (http://www.ncbi.nlm.nih.gov/geo/) to further validate the expression of ARNTL2 in PDAC samples and normal tissues. The characteristics of the datasets were showed in Supplemental Table S1.

### Cell culture

The PDAC cell lines (BXPC-3, CFPAC-1, SW1990, PANC-1), embryonic kidney cell lines (HEK293) and normal pancreatic duct cell lines (HPDEC) were purchased from the Chinese Academy of Science (Shanghai, China). Cells were cultured as previous described^29^, Cells were maintained in Dulbecco’s modified Eagle’s medium (DMEM) supplemented with 10% fetal bovine serum (FBS) (Gibco, NY, USA) and 100 U/ml penicillin/streptomycin (Corning, NY, USA) in a humidified incubator under a 5% CO_2_ atmosphere at 37 °C. The detail of these cells was shown in Supplemental Table S2.

### Immunohistochemistry staining

Tissue microarrays (TMA) consisted of 96 PDAC specimens and their matched nonneoplastic counterparts (ZZU cohort). The IHC staining was performed to investigate ARNTL2 expression in TMA. Based on the different percentage of positively stained cells and staining intensity, we established a semi-quantitatively scoring system as previously described^30^, the staining extent in each core was scored as 1+ (<25% staining of tumor cells), 2+ (25–50% staining of tumor cells), 3+ (50–75% staining of tumor cells), or 4+ (>75% staining of tumor cells). In addition, the staining intensity was quantified as 0 (negative), 1+ (weak), 2+ (intermediate), or 3+ (strong). The final immunoreaction score was obtained by multiplying the intensity and extension values (range 0–12) and the samples were grouped as 1+ (score 0), 2+ (score 1–2), 3+ (score 3–4), 4+ (score 6–8), and 5+ (score 9–12) staining. Meanwhile, for statistical purposes, scores of 4+ and 5+ were defined as high expression and the other final scores were considered as low expression. Supplemental Table S3 listed the antibody information used in this study.

### Western blotting

Total proteins were prepared using RIPA lysis buffer (Beyotime, China). Proteins were quantified by BCA assay. Equal amount of proteins was loaded to gel and separated by SDS-PAGE and electrotransferred onto polyvinylidene difluoride membranes. The members were blocked using 5% non-fat milk for 1 h at room temperature. The membranes were incubated with primary antibodies at 4 °C overnight. Then the members were probed with the corresponding secondary antibodies for 1 h at 37 °C. The band was visualized by enhanced Luminol/Enhancer (Bio‐Rad) chemiluminescence (Bio-Rad, USA). Supplemental Table S3 listed the information of antibodies.

### RNA extraction and quantitative RT-PCR (qRT-PCR)

Total RNA from CFPAC-1 and PANC-1 cells was extracted using the TRIzol Reagent (Life Technologies Inc., Carlsbad, CA, USA), as instructed by the manufacturer. RNA was used for cDNA synthesis with the Superscript III Reverse Transcription Reagent (Life Technologies). qRT-PCR analysis with SYBR Green was performed on the total RNA extracted from cell lines and tissues by means of an ABI7500 real-time PCR detection system (Applied Biosystems, Foster City, CA, USA). β-Actin or U6 small nuclear RNA served as an endogenous control. Each assay was conducted in triplicate and the 2 − ΔΔCt method was used to calculate relative expression.

### Cell proliferation and colony formation assays

In order to compare the cell growth rate, 3 × 10^3^ μL cells were inoculated in 96-well plate in 100 μL medium and cultured overnight. Cells growth was evaluated using the Cell Counting Kit-8 (CCK-8) Test (Beyotime, China) and Edu assay kit (Ribobio, Guangzhou, China). For colony formation assays, the number of colonies (defined as containing more than 50 cells) was determined under a light microscopy.

### In vitro migration and invasion assays

Wound-healing assay was conducted to determine the migration ability. CFPAC-1 and PANC-1 cells (5 × 10^6^) were seeded into six-well plates. A 1-mm wide wound was created using a 200-μL sterile tip after 90% confluence was reached. The wounded areas were observed and photographed every 24 h under microscope.

Cell invasion was tested by Transwell assay. The logarithmic CFPAC-1 and PANC-1 cells were collected, adjusted to 5 × 10^4^/ml, and then added into the upper chamber of Transwell chamber. A complete medium containing 10% fetal bovine serum was added to the lower chamber. After 24 h incubation, Transwell chamber was removed, washed, fixed with 4% paraformaldehyde, and stained with crystal violet for 20 min. The number of invasive cells was counted under an inverted optical microscope, and the average number of cells in each field was counted

### Luciferase assays

Wild-type and mutated 3′-UTR fragments of ARNTL2 mRNA containing the predicted miR-26a-5p-binding site were amplified and cloned into the psiCHECK-2 luciferase reporter vector (Promega, Madison, WI, USA). HEK293 Cells were co-transfected with miR-26a-5p mimics or NC control, together with either ARNTL2-wt or ARNTL2-mut reporter vectors. Twenty-four hours later, the luciferase intensity was measured and normalized to the renilla luciferase intensity using the Dual-Luciferase^®^ Reporter Assay System (Promega) according to the manufacturer’s instructions.

### Lentiviral transduction and vector construction

ARNTL2-knockdown shRNA or overexpression vectors, miR-26a-5p mimics, and inhibitors were obtained from Hanbio Company (Shanghai, China). CFPAC-1 and PANC-1 cells (1 × 10^5^) were transduced with lentivirus encoding miR-26a-5p mimics, miR-26a-5p inhibitor, ARNTL2-overexpression plasmid, ARNTL2 shRNA, or an empty lentivirus for 96 h, and then selected by puromycin treatment (Santa Cruz Biotechnology, CA, USA) for 4 weeks.

### Tumor growth and tumor metastasis experiments in nude mice

To establish the nude mice models of PDAC, PANC-1 cell lines transfected with ARNTL2-knockdown lentivirus (sh-ARNTL2), miR-26a-5p mimics (Lenti-miR-26a-5p), and empty lentivirus control (NC) were implanted into the flank of the nude mice (1 × 10^7^ cells/mice in 100 μl PBS, *n* = 6 per group). Tumor growth was measured after 1 week, and tumor volumes were calculated by the following formula: volume (cm^3^) = (length × width^2^)/2. After 4 weeks, the mice were sacrificed and the tumors were collected and weighed, and tissue sections were obtained for further IHC staining. For the tumor metastasis experiment, nude mice were subjected to tail vein injection of PANC-1 cells stably knock down ARNTL2 (sh-ARNTL2) or empty lentivirus control (NC). At 35 day, all the mice were sacrificed and the lung were excised, and the number of metastatic nodules in the lung was counted. All animal experiments were approved by the Animal Care Committee of the First Affiliated Hospital of Zhengzhou University.

### Statistical analysis

Statistical analysis was conducted using SPSS V23.0 (IBM, Armonk, NY, USA). The Student’s *t* test was used to analyze the difference between two groups. Pearson *χ*^2^ tests were adopted to analyze the association of ARNTL2 expression with clinicopathological variables. The survival analysis was performed using the Kaplan–Meier method, and differences in survival curves were evaluated by the log-rank test. Univariate and multivariate cox regression models were performed to determine independent prognostic factors. The correlation was performed by Pearson correlation test. A *P* value of <0.05 was considered statistically significant.

## Supplementary information

Supplementary Figure legends clean version

Supplementary Figure S1

Supplementary Figure S2

Supplementary Figure S3

Supplementary Figure S4

Supplementary table S1-S3
