# Supplementary material for: ARNTL2 promotes pancreatic ductal adenocarcinoma progression through TGF/BETA pathway and is regulated by miR-26a-5p
Source: Cell Death Dis. 2020 Aug 10;11(8):692. doi: 10.1038/s41419-020-02839-6 (PMC7443143; doi:10.1038/s41419-020-02839-6)
Supplement: Supplementary file 1 — Supplementary Figure legends clean version [file 41419_2020_2839_MOESM1_ESM.docx]

**Fig S1. Downregulation of ARNTL2 promotes PDAC cell apoptosis *in vitro*.** (**A**) The protein expression levels of ARNTL2 in normal pancreas cells (HPDEC) and PDAC cell lines (BXPC-3, CFPAC-1, SW1990 and PANC-1) were determined by western blot analysis. (**B**) Confirmation of ARNTL2 knockdown in PDAC cell lines by immunoﬂuorescence. Scale bars, 50μm. (**C**) Tunel assay demonstrated that ARNTL2-knockdown promoted PDAC cell apoptosis rate. Scale bars, 50μm. (**D**) Apoptosis related proteins were analyzed by western blot. **p* < 0.05, ***p* < 0.01, ****p* < 0.001.

**Fig S2. Ectopic expression of ARNTL2 promotes PDAC cell proliferation and metastasis.** (**A**) Western blot was performed to confirm the transfection efficiency. (**B-E**) Cell proliferation capacity was analyzed by CCK-8 assay (**B, C**), EDU staining assay, Scale bars, 50μm (**D**) and colony formation assay, Scale bars, 8mm (**E**). The above functional experiments revealed that ectopic expression of ARNTL2 dramatically promoted PDAC cells proliferation, DNA synthesis and colony formation. **(F)** Wound healing assay showed that ARNTL2 elevated expression could promote the migration capacity of PDAC cells. **(G)** The invasion capability of PDAC cells transfected with NC or ARNTL2 plasmid was analyzed by transwell assay. Scale bars, 50μm. **p* < 0.05, ***p* < 0.01, ***p* < 0.001.

**Fig S3. Enforced expression of miR-26a-5p represses tumor growth in PDAC xenografts.** (**A**) Tumor weight were lower in Lenti-miR-26a-5p group compared with those in NC group**.** (**B**) Representative H&E staining and Ki-67, ARNTL2 IHC staining in PDAC xenografts after Lenti-miR-26a-5p treatment. Scale bars, 200 μm. (**F**) Relative IHC expression levels of ARNTL2 and Ki67 were obviously decreased in Lenti-miR-26a-5p group compared with the control group (NC). ***p* < 0.01, ****p* < 0.001

**Fig S4. ARNTL2 regulates PDAC cell growth, migration and invasion via targeting TGF-β1.** CFPAC-1 or PANC-1 cells were transfected with NC, ARNTL2 plasmid, ARNTL2 plasmid & si-TGF-β1. Cell proliferation was assessed by colony formation assay (A). Cell invasion ability was analyzed by transwell assay. Scale bars, 50 μm (B). Cell migration capability was analyzed by wound-healing assay. Scale bars, 500μm (C). The above functional experiments certified that the enhancing effect of ARNTL2 plasmid on PDAC cell growth could be partially abolished by si-TGF-β1. Subsequently, CFPAC-1 or PANC-1 cells were transfected with NC, si-ARNTL2, si-ARNTL2 & TGF-β1 plasmid. The inhibitory effect of si-ARNTL2 on PDAC cell colony could be partially abolished by TGF-β1 plasmid (D). Consistent results were observed in transwell assay (Scale bars, 50 μm) (E) and wound-healing assay (Scale bars, 500μm) (F).
