# Supplementary figures and images for: ARNTL2 promotes pancreatic ductal adenocarcinoma progression through TGF/BETA pathway and is regulated by miR-26a-5p
Source: Cell Death Dis. 2020 Aug 10;11(8):692. doi: 10.1038/s41419-020-02839-6 (PMC7443143; doi:10.1038/s41419-020-02839-6)

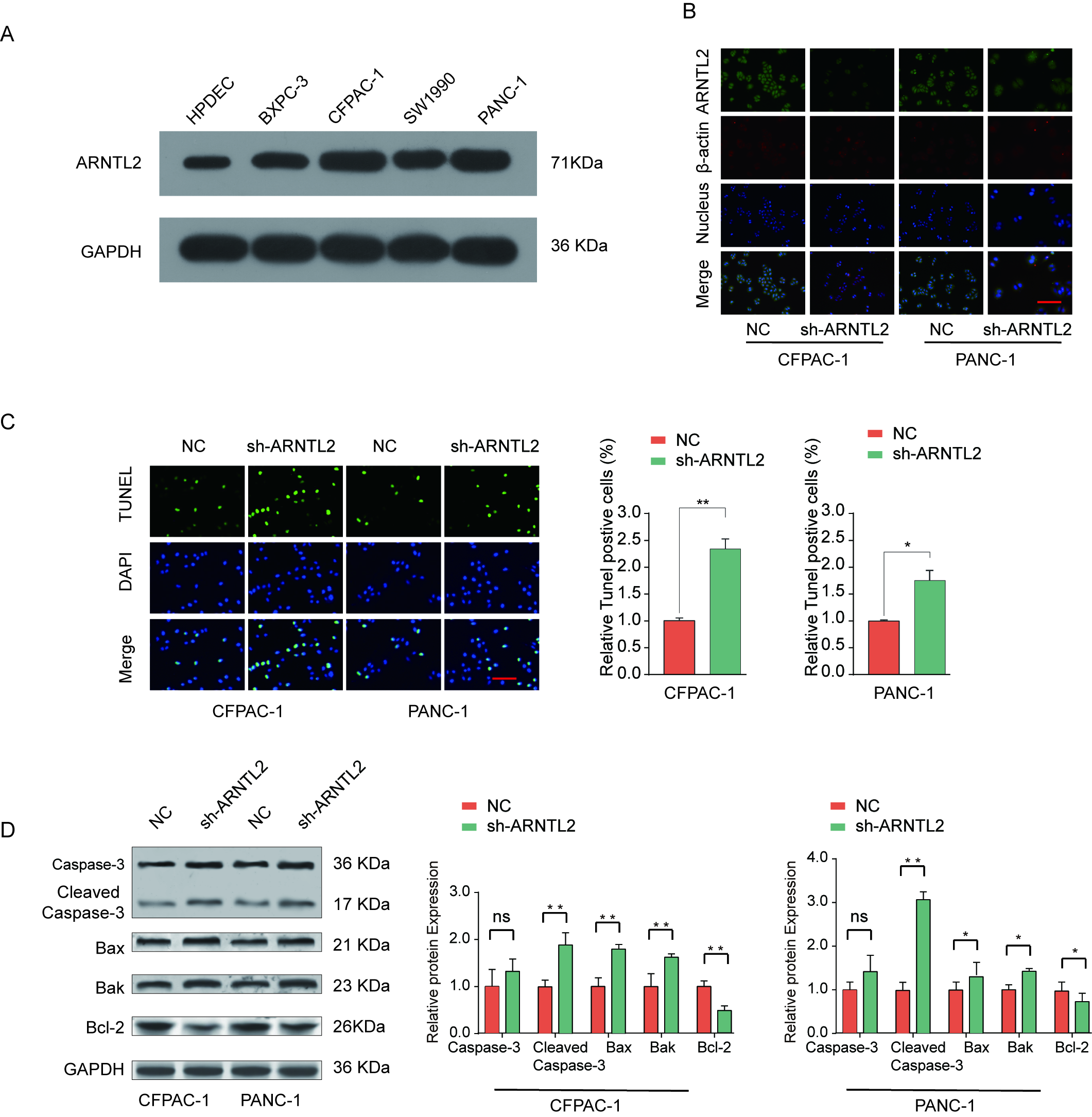

Supplement: Supplementary file 2 — Supplementary Figure S1 [file 41419_2020_2839_MOESM2_ESM.tif]

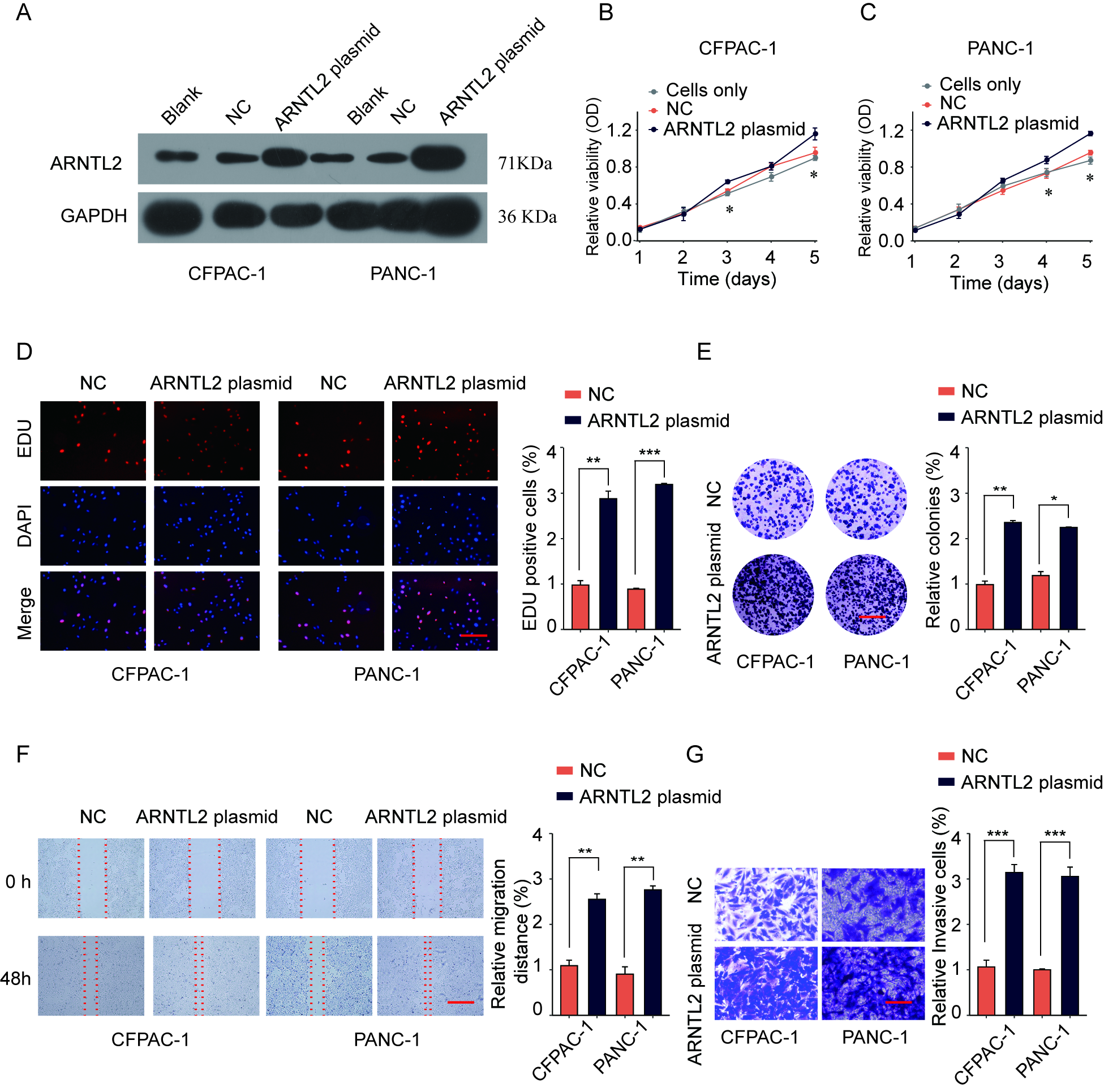

Supplement: Supplementary file 3 — Supplementary Figure S2 [file 41419_2020_2839_MOESM3_ESM.tif]

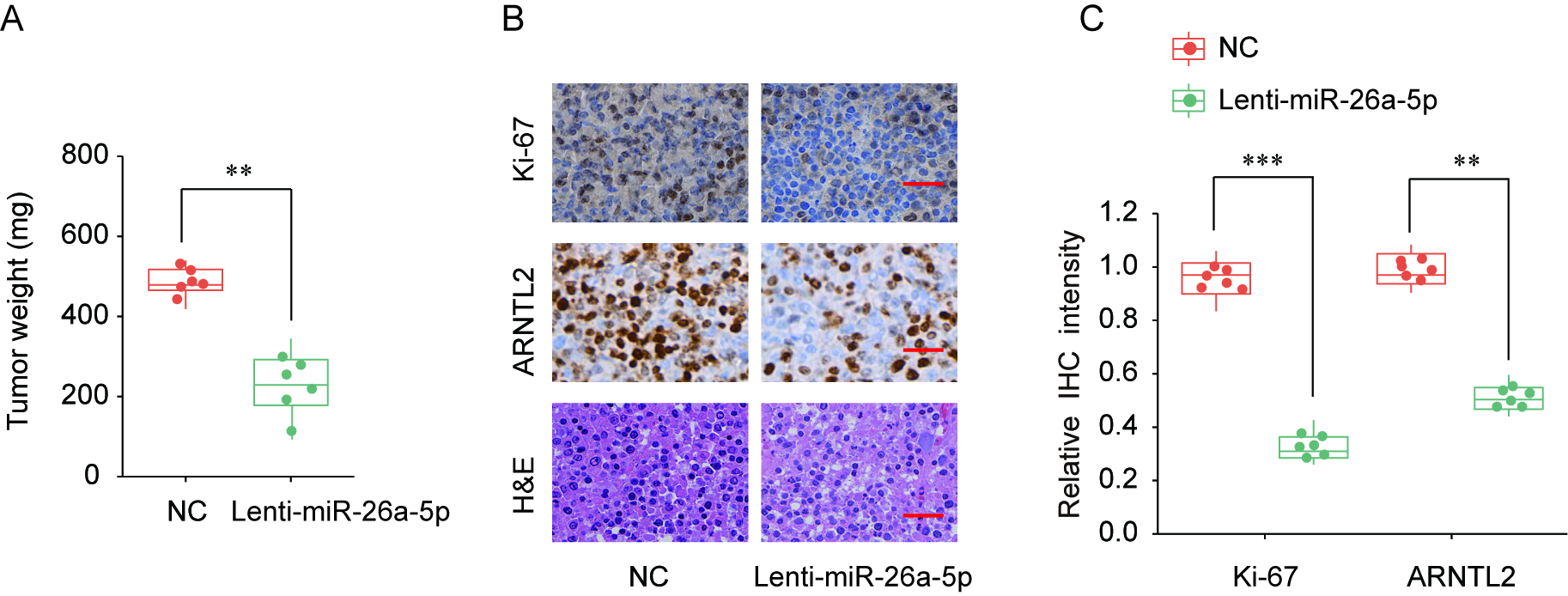

Supplement: Supplementary file 4 — Supplementary Figure S3 [file 41419_2020_2839_MOESM4_ESM.tif]

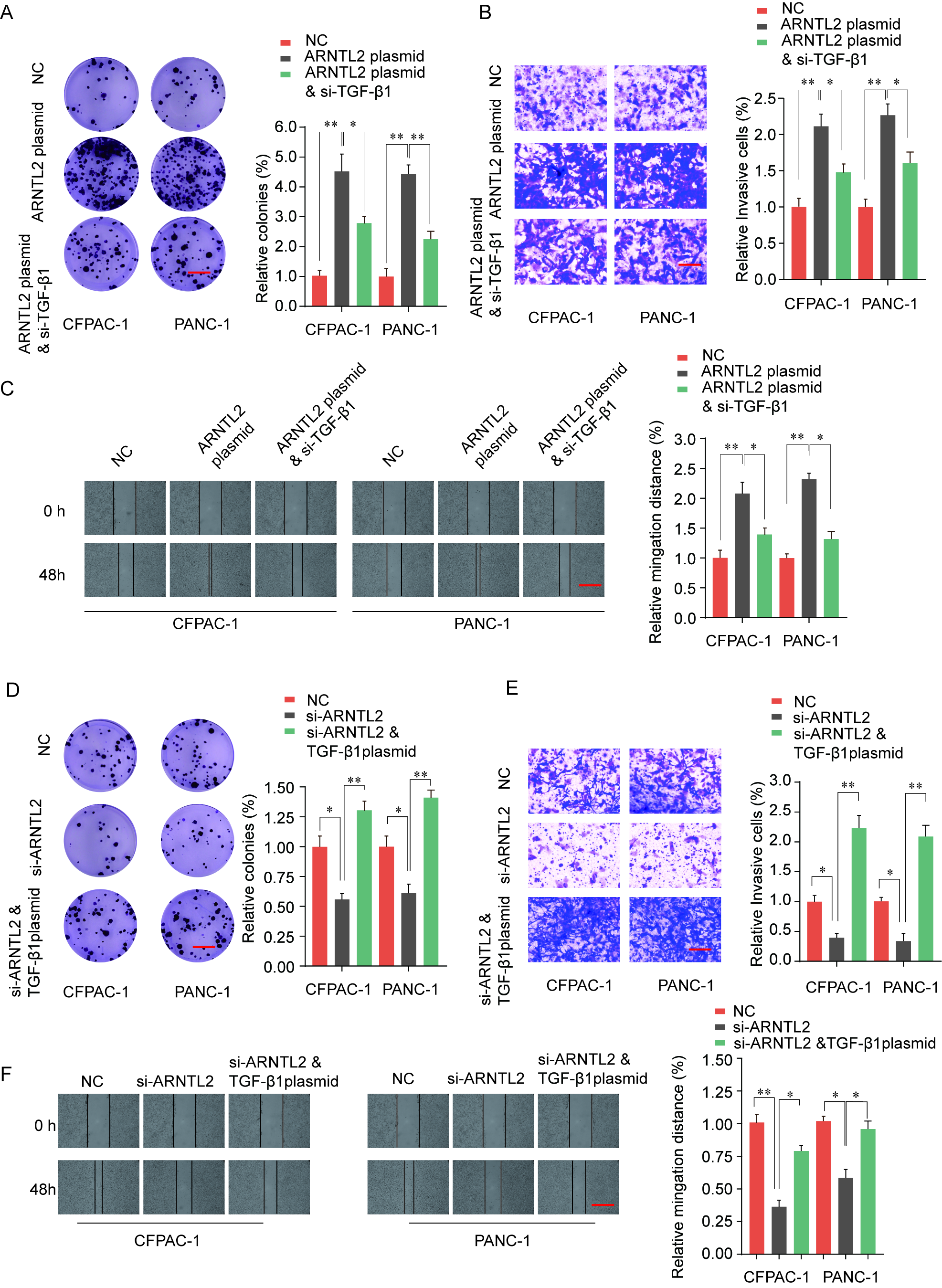

Supplement: Supplementary file 5 — Supplementary Figure S4 [file 41419_2020_2839_MOESM5_ESM.tif]
