# Supplementary material for: ARNTL2 promotes pancreatic ductal adenocarcinoma progression through TGF/BETA pathway and is regulated by miR-26a-5p
Source: Cell Death Dis. 2020 Aug 10;11(8):692. doi: 10.1038/s41419-020-02839-6 (PMC7443143; doi:10.1038/s41419-020-02839-6)
Supplement: Supplementary file 6 — Supplementary table S1-S3 [file 41419_2020_2839_MOESM6_ESM.docx]

**Supplementary Table S1. GEO information used in this study**

| GEO ID | Platforms | Non-tumor | Tumor | Year | Country |
| --- | --- | --- | --- | --- | --- |
| GSE102238 | Affymetrix mRNA microarray | 50 | 50 | 2017 | China |
| GSE15471 | Affymetrix mRNA microarray | 39 | 39 | 2009 | Romania |
| GSE16515 | Affymetrix mRNA microarray | 36 | 36 | 2009 | USA |
| GSE28735 | Affymetrix mRNA microarray | 45 | 45 | 2011 | USA |
| GSE55643 | Affymetrix mRNA microarray | 8 | 45 | 2014 | United Kingdom |
| GSE62165 | Affymetrix mRNA microarray | 13 | 118 | 2014 | Belgium |
| GSE62452 | Affymetrix mRNA microarray | 61 | 69 | 2014 | USA |
| GSE71729 | Affymetrix mRNA microarray | 134 | 223 | 2015 | USA |
| GSE73338 | Affymetrix mRNA microarray | 9 | 88 | 2015 | United Kingdom |
| Total |  | **395** | **713** |  |  |

**Supplementary Table S2. Cell lines used in this study**

| **Cell lines** | **Cell type** | **Source** | **Country** |
| --- | --- | --- | --- |
| BXPC-3 | Pancreatic cancer cell | Cell Bank of the Chinese  Academy of Science | China |
| CFPAC-1 | Pancreatic cancer cell | Cell Bank of the Chinese  Academy of Science | China |
| SW1990 | Pancreatic cancer cell | Cell Bank of the Chinese  Academy of Science | China |
| PANC-1 | Pancreatic cancer cell | Cell Bank of the Chinese  Academy of Science | China |
| HPDEC | Normal pancreatic duct cell | Cell Bank of the Chinese  Academy of Science | China |
| HEK293 | Embryonic kidney cell | Cell Bank of the Chinese  Academy of Science | China |

**Supplementary Table S3. Information on antibodies used in this study**

| **Antibody** | **WB** | **IHC** | **Specificity** | **Company** |
| --- | --- | --- | --- | --- |
| GAPDH | 1:20000 | / | Mouse monoclonal | Proteintech Group, China |
| ARNTL2 | 1:500 | 1:500 | Rabbit Polyclonal | Proteintech Group, China |
| Ki-67 | / | 1:500 | Rabbit Polyclonal | Proteintech Group, China |
| TGF-β1 | 1:500 | 1:500 | Rabbit Polyclonal | Proteintech Group Chicago, USA |
| BMP4 | 1:500 | 1:200 | Rabbit Polyclonal | Proteintech Group Chicago, USA |
| ICAM1 | 1:500 | 1:500 | Rabbit Polyclonal | Proteintech Group Chicago, USA |
| VCAM1 | 1:500 | 1:400 | Rabbit Polyclonal | Proteintech Group Chicago, USA |
